# Antimicrobial Activity and DNA/BSA Binding Affinity of Polynuclear Silver(I) Complexes with 1,2-Bis(4-pyridyl)ethane/ethene as Bridging Ligands

**DOI:** 10.1155/2020/3812050

**Published:** 2020-04-14

**Authors:** Sonja Ž. Đurić, Sandra Vojnovic, Tina P. Andrejević, Nevena Lj Stevanović, Nada D. Savić, Jasmina Nikodinovic-Runic, Biljana Đ. Glišić, Miloš I. Djuran

**Affiliations:** ^1^University of Kragujevac, Faculty of Science, Department of Chemistry, R. Domanovića 12, 34000 Kragujevac, Serbia; ^2^Institute of Molecular Genetics and Genetic Engineering, University of Belgrade, Vojvode Stepe 444a, 11000 Belgrade, Serbia; ^3^University of Kragujevac, Institute for Information Technologies Kragujevac, Department of Science, Jovana Cvijića bb, 34000 Kragujevac, Serbia; ^4^Serbian Academy of Sciences and Arts, Knez Mihailova 35, 11000 Belgrade, Serbia

## Abstract

1,2-Bis(4-pyridyl)ethane (bpa) and 1,2-bis(4-pyridyl)ethene (bpe) were used for the synthesis of polynuclear silver(I) complexes, {[Ag(bpa)]NO_3_}_n_ (**1**), {[Ag(bpa)_2_]CF_3_SO_3_^.^H_2_O}_n_ (**2**) and {[Ag(bpe)]CF_3_SO_3_}_n_ (**3**). In complexes **1–3**, the corresponding nitrogen-containing heterocycle acts as a bridging ligand between two Ag(I) ions. *In vitro* antimicrobial activity of these complexes, along with the ligands used for their synthesis, was evaluated against the broad panel of Gram-positive and Gram-negative bacteria and fungi. The silver(I) complexes **1–3** showed selectivity towards *Candida* spp. and Gram-negative *Escherichia coli* in comparison to the other investigated bacterial strains, effectively inhibiting the growth of four different *Candida* species with minimal inhibitory concentrations (MICs) between 2.5 and 25 *μ*g/mL and the growth of *E. coli*, with MIC value being 12.5 *μ*g/mL. Importantly, complex **2** significantly reduced *C. albicans* filamentation, an essential process for its pathogenesis. Antiproliferative effect on the normal human lung fibroblast cell line MRC-5 was also evaluated with the aim of determining the therapeutic potential of the complexes **1–3**. The interactions of these complexes with calf thymus DNA (ctDNA) and bovine serum albumin (BSA) were studied to evaluate their binding activities towards these biomolecules for possible insights on their mode of action.

## 1. Introduction

The invasive microbial infections are seen as a rapidly increasing global threat to human health, in particular in immunocompromised patients, having an unacceptably high mortality rate despite the availability of antimicrobial drugs [1, 2]. This high mortality rate originates primarily from an inadequate diagnostics and shortcomings of the conventionally used agents, such as toxic side effects and/or resistance development. Compared to the traditional organic (synthetic or natural) drugs, metal-containing therapeutics might have the advantages in the synergistic effect, the accessible redox states, and the tunable pharmacophore geometries [3, 4]. Since the successful use of cream containing silver(I) sulfadiazine for the treatment of burn wounds [5], numerous silver(I) complexes have been synthesized and screened for their antimicrobial properties. Silver(I) complexes showed effective and wide-spectrum antimicrobial activity, including the strains which are resistant to the currently used antimicrobials, while their toxicity to the normal human cells was not pronounced [6]. Besides that, one of the main advantages of silver(I) complexes, in comparison to the used antimicrobials, is their multidirectional activity, which slows down the evolution of resistance [7]. Silver(I)-containing compounds may interact with the cell wall and, once inside the cell, they interact with the biomolecules in its interior, such as DNA and proteins [7]. Previously, it has been reported that the bacterial cells treated with Ag(I) ions developed a region with condensed DNA, which lost the ability to replicate [8]. On molecular level, Ag(I) ion is known to bind to DNA nitrogen bases, with guanine and adenine N7 atoms being its preferential binding sites [9]. This binding has resulted in alternation of the normal DNA transcription regulation disrupting genome and cellular processes [10, 11]. Moreover, the mechanism of antimicrobial activity of Ag(I) ion includes its interaction with the thiol group of L-cysteine residue of proteins and consequent enzyme inhibition and the generation of intracellular reactive oxygen species [7].

The crucial factors in determining the antimicrobial effectiveness of silver(I) complexes are the type of the ligand donor atom bound to the Ag(I) ion and the strength of the resulting Ag–donor bonds [12]. Thus, silver(I) complexes with various aromatic nitrogen-donor ligands have shown remarkable and broad-spectrum antimicrobial activity against a panel of bacterial and fungal strains as a consequence of a relatively weak Ag–N bond which can be easily cleaved in the reactions with biological target molecules [13–21]. As a continuation of our efforts in the synthesis of silver(I) complexes as potential antimicrobial agents, in the present study, we used two aromatic nitrogen-containing heterocycles (*N*-heterocycles) in which two pyridine rings are linked by–CH_2_–CH_2_– and–CH**═**CH–groups, 1,2-bis(4-pyridyl)ethane (bpa) and 1,2-bis(4-pyridyl)ethene (bpe), respectively. These ligands have previously been used as linkers in the synthesis of metal-organic frameworks [22, 23]. Considering the global shortage of efficient antimicrobials to successfully keep the combat against drug-resistant pathogens, it is important to consider the wealth of previously reported structures as new untested chemical diversity that can potentially deliver new antibiotics. Therefore, starting from AgX salt (X = NO_3_^−^ and CF_3_SO_3_^−^) and the abovementioned *N*-heterocycles, three polynuclear silver(I) complexes, {[Ag(bpa)]NO_3_}_n_ (**1**), {[Ag(bpa)_2_]CF_3_SO_3_^.^H_2_O}_n_ (**2**), and {[Ag(bpe)]CF_3_SO_3_}_n_ (**3**), were synthesized using slightly different approach. The complexes were evaluated for their *in vitro* antimicrobial activity and the effect on the viability of a human normal fibroblast cell line (MRC-5). In order to gain an insight into the reactivity of complexes **1–3** with potential biological targets, their interactions with the calf thymus DNA (ctDNA) and bovine serum albumin (BSA) were examined.

## 2. Materials and Methods

### 2.1. Materials

The silver(I) salts (AgNO_3_ and AgCF_3_SO_3_), 1,2-bis(4-pyridyl)ethane (bpa), 1,2-bis(4-pyridyl)ethene (bpe), ethanol, acetonitrile, dimethyl sulfoxide (DMSO), deuterated dimethyl sulfoxide (DMSO-*d*_6_), calf thymus DNA (ctDNA), Tris buffer (tris(hydroxymethyl)aminomethane), ethidium bromide (EthBr), and bovine serum albumin (BSA) were obtained from the Sigma-Aldrich. All chemicals were of reagent-grade quality or higher and used without further purification.

### 2.2. Measurements

The elemental analyses of the synthesized silver(I) complexes for carbon, hydrogen, and nitrogen were performed by the Microanalytical Laboratory, Faculty of Chemistry, University of Belgrade. The ^1^H and ^13^C NMR spectra of the *N*-heterocyclic ligands and the silver(I) complexes were recorded at room temperature on a Varian Gemini 2000 spectrometer (^1^H at 200 MHz, ^13^C at 50 MHz). 5.0 mg of each compound was dissolved in 0.6 mL of DMSO-*d*_6_ and transferred into a 5 mm NMR tube. Chemical shifts, *δ*, are reported in parts per million (ppm) and scalar couplings (*J*) are reported in Hertz. Chemical shifts were calibrated relative to those of the solvent. The abbreviations for the peak multiplicities are as follows: *s* (singlet), *d* (doublet), dd (doublet of doublets), and *m* (multiplet). In order to investigate the solution behavior of silver(I) complexes, the ^1^H NMR spectra were recorded immediately after their dissolution and after 48 h standing in the dark at room temperature. The UV-Vis spectra were recorded on a Shimadzu double-beam spectrophotometer after dissolving the corresponding silver(I) complex in DMSO over the wavelength range of 200–900 nm. The concentration of the silver(I) complexes was 2.36·10^−4^ (**1**), 4.31^.^10^−5^ (**2**), and 1.50^.^10^−5^ M (**3**). The IR spectra were recorded as KBr pellets on a PerkinElmer Spectrum 100 spectrometer over the wavenumber range of 450–4000 cm^−1^.

### 2.3. Synthesis of the Silver(I) Complexes **1**–**3**

The method applied for the preparation of these complexes has been optimized in comparison to the previous reports [24, 25] and resulted in a high yield complex formation.

The solution of 1.0 mmol of the corresponding silver(I) salt (169.9 mg of AgNO_3_ for **1** and 256.9 mg of AgCF_3_SO_3_ for **2** and **3**) in 5.0 mL of ethanol was added slowly under stirring to the solution containing 0.5 mmol of 1,2-bis(4-pyridyl)ethane (bpa; 92.1 mg for **1** and **2**) and 1,2-bis(4-pyridyl)ethene (bpe; 91.1 mg for **3**) in 5.0 mL of ethanol. A white precipitate was formed immediately after addition of the silver(I) salt. The reaction mixture was stirred in the dark at ambient temperature for 4 h, and then, the precipitate was filtered off and recrystallized in acetonitrile (**1**) or acetonitrile/water (v/v 1 : 1; **2** and **3**). The obtained solutions were left at room temperature, and after 4–6 days, colorless crystals of complexes **1–3** were formed. These crystals were filtered off and dried in the dark at ambient temperature. Yield (calculated on the basis of the *N*-heterocyclic ligand) was 132.8 mg (75%) for **1**, 114.2 mg (71%) for **2**, and 184.4 mg (84%) for **3**.

Elemental analysis for **1** = C_12_H_12_AgN_3_O_3_ (MW = 354.12): found: C, 40.54; H, 3.51; and N, 11.69%; calc.: C 40.70; H, 3.42; and N, 11.87%. ^1^H NMR (200 MHz, DMSO-*d*_6_): *δ* = 2.98 (s, CH_2_), 7.32 (d, *J* = 5.8 Hz, H3 and H5), and 8.46 (s, H2 and H6) ppm. ^13^C NMR (50 MHz, DMSO-*d*_6_): *δ* = 34.57 (CH_2_), 124.31 (C3 and C5), 149.36 (C2 and C6), and 150.59 (C4) ppm. IR (KBr, *ν*, cm^−1^): 3030 w, 2925 w (*ν*(C_ar_–H)), 2858w (*ν*(C–H)), 1606 s, 1559 w, 1499 w (*ν*(C_ar_ = C_ar_) and *ν*(C_ar_ = N)), 1384 (*ν*_as_(NO_3_)), 1221 m (*ν*(C_ar_–N)), 826 s, 546 m (*γ*(C_ar_–H)). UV-Vis (DMSO, λ_max_, nm): 257 (*ε* = 3.3·10^3^ M^−1^·cm^−1^).

Elemental analysis for **2** = C_25_H_26_AgF_3_N_4_O_4_S (MW = 643.43): found: C, 46.44; H, 4.01; and N, 8.59%; calc.: C 46.67; H, 4.07; and N, 8.71%. ^1^H NMR (200 MHz, DMSO-*d*_6_): *δ* = 2.96 (s, CH_2_), 7.29 (dd, *J* = 4.5, 1.5 Hz, H3 and H5), 8.45 (dd, *J* = 4.5, 1.5 Hz, H2 and H6) ppm. ^13^C NMR (50 MHz, DMSO-*d*_6_): *δ* = 34.52 (CH_2_), 124.05 (C3 and C5), 149.57 (C2 and C6), 150.02 (C4) ppm. IR (KBr, *ν*, cm^−1^): 3429br (*ν*(O–H)), ∼3000 w (*ν*(C_ar_–H)) and (*ν*(C–H)), 1615 s, 1560 m, 1507 w, 1434 m (*ν*(C_ar_ = C_ar_) and *ν*(C_ar_ = N)), 1279vs, 1251vs (*ν*_as_(SO_3_)), 1223 s (*ν*_s_(CF_3_)), 1160 s (*ν*_as_(CF_3_)), 1024vs (*ν*_s_(SO_3_)), 831 m, 597 w (*γ*(C_ar_–H)). UV-Vis (DMSO, *λ*_max_, nm): 257 (*ε* = 1.8·10^4^ M^−1^·cm^−1^).

Elemental analysis for **3** = C_13_H_10_AgF_3_N_2_O_3_S (MW = 439.16): found: C, 35.32; H, 2.11; and N, 6.29%; calc.: C 35.55; H, 2.30; and N, 6.38%. ^1^H NMR (200 MHz, DMSO-*d*_6_): *δ* = 7.57 (s, CH), 7.65 (dd, *J* = 4.6, 1.7 Hz, H3 and H5), 8.61 (dd, *J* = 4.6, 1.6 Hz, H2 and H6) ppm. ^13^C NMR (50 MHz, DMSO-*d*_6_): *δ* = 121.34 (C3 and C5), 130.66 (CH), 143.51 (C4), 150.23 (C2 and C6) ppm. IR (KBr, *ν*, cm^−1^): 3061 w, 2921 w (*ν*(C–H)), 1610 s, 1560 m, 1505 m, 1434 m (*ν*(C=C) and *ν*(C_ar_ = N)), 1280vs, 1251vs (*ν*_as_(SO_3_)), 1222 s (*ν*_s_(CF_3_)), 1156vs (*ν*_as_(CF_3_)), 1025vs (*ν*_s_(SO_3_)), 837 m, 553 m (*γ*(C_ar_–H)). UV-Vis (DMSO, *λ*_max_, nm): 303 (*ε* = 4.9·10^4^ M^−1^·cm^−1^) and 313 (shoulder, (*ε* = 4.0·10^4^ M^−1^·cm^−1^).

bpa data given for comparative purposes: MW = 184.24. ^1^H NMR (200 MHz, DMSO-*d*_6_): *δ* = 2.94 (s, CH_2_), 7.25 (dd, *J* = 4.4, 1.6 Hz, H3 and H5), 8.44 (dd, *J* = 4.4, 1.6 Hz, H2 and H6) ppm. ^13^C NMR (50 MHz, DMSO-*d*_6_): *δ* = 34.56 (CH_2_), 123.86 (C3 and C5), 149.39 (C2 and C6), 149.64 (C4) ppm. IR (KBr, *ν*, cm^−1^): 3030 m, 2947 m, 2926 m, 2859 m, (*ν*(C_ar_–H)) and (*ν*(C–H)), 1595 s, 1558 m, 1493 w, 1455 m, 1414 s (*ν*(C_ar_ = C_ar_) and *ν*(C_ar_ = N)), 828vs, 807 m, 546vs, 516 m (γ(C_ar_–H)). UV-Vis (DMSO, λ_max_, nm): 258 (*ε* = 4.4·10^3^ M^−1^·cm^−1^).

bpe data given for comparative purposes: MW = 182.22. ^1^H NMR (200 MHz, DMSO-*d*_6_): *δ* = 7.53 (s, CH), 7.60 (dd, *J* = 4.5, 1.6 Hz, H3 and H5), 8.60 (dd, *J* = 4.5, 1.6 Hz, H2 and H6) ppm. ^13^C NMR (50 MHz, DMSO-*d*_6_): *δ* = 121.23 (C3 and C5), 130.56 (CH), 143.32 (C4), 150.14 (C2 and C6) ppm. IR (KBr, *ν*, cm^−1^): 3101 w, 3021 m, 2985 w, 2890 w (*ν*(C–H)), 1595vs, 1552 m, 1496 m, 1410 s (*ν*(C=C) and *ν*(C_ar_ = N)), 835 m, 820 s, 552 m, 535 m (*γ*(C_ar_–H)). UV-Vis (DMSO, *λ*_max_, nm): 303 (*ε* = 2.1·10^4^ M^−1^·cm^−1^) and 315 (*ε* = 1.9·10^4^ M^−1^·cm^−1^).

### 2.4. Antimicrobial Susceptibility Testing

Stock solutions of bpa, bpe, and silver(I) complexes **1–3** were prepared fresh in DMSO (50 mg/mL) and kept at 4°C prior to use. Susceptibility testing of *Candida* spp. (*C. albicans* ATCC 10231, *C. krusei* ATCC 6258, *C. parapsilosis* ATCC 22019, and *C. glabrata* ATCC 2001) was performed according to CLSI broth microdilution guidelines (Clinical and Laboratory Standards Institute, Reference Method for Broth Dilution Antifungal Susceptibility Testing of Yeasts—Third Edition: Approved Standard M27-A3; Clinical and Laboratory Standards Institute, Reference Method for Broth Dilution Antifungal Susceptibility Testing of Yeasts: Fourth Informational Supplement M27-S4), in RPMI 1640 medium (Roswell Park Memorial Institute medium) containing 2% glucose (w/v). The highest tested concentration was 500 *μ*g/mL, and the inocula were 1^·^\10^5^ colony forming units (cfu)/mL.

Minimal inhibitory concentration (MIC) values against *Pseudomonas aeruginosa* PA01, *Staphylococcus aureus* ATCC 43300, *Salmonella enteritidis* ATCC 13075, *Salmonella pullorum* ATCC 13036, *Escherichia coli* NCTC 9001, *Listeria monocytogenes* NCTC 11994, and *Enterococcus faecalis* ATCC 29212 were determined in Luria-Bertani broth (10.0 g/L tryptone, 10.0 g/L NaCl, 5.0 g/L yeast extract, pH 7.2) in accordance with the standard broth microdilution assay for bacteria that grow aerobically, as recommended by the CLSI (Clinical and Laboratory Standards Institute, Methods for Dilution Antimicrobial Susceptibility Tests for Bacteria That Grow Aerobically; Approved Standard—Tenth Edition M07-A10. CLSI). The highest tested concentration was 500 *μ*g/mL, and the inocula were 1^·^10^6^ cfu/mL. MIC values were read after 24 h of incubation at 37°C as the lowest concentration to exhibit absence of growth.

### 2.5. C. albicans Filamentation Study

The suspension of *C. albicans* expressing red fluorescent protein was prepared in RPMI 1640 medium with 10% (v/v) fetal bovine serum (FBS) to induce hyphae formation [26]. The suspension was then treated with MIC_80_ concentrations of **1–3** at 37°C shaking. Cells treated with DMSO served as the control. After 6 h of incubation, the results were observed by a fluorescence microscope (Olympus BX51, Applied Imaging Corp., San Jose, CA, United States) under 20× magnification.

### 2.6. MTT Assay on a Human Fibroblast Cell Line

Antiproliferative activity was tested by 3-(4,5-dimethylthiazol-2-yl)-2,5-diphenyltetrazolium bromide (MTT) assay on a human lung fibroblast cell line (MRC-5; ATCC collection) [27]. Pregrown (24 h) cell monolayers were incubated in the media containing the tested compounds at concentrations ranging from 5 to 500 *μ*g/mL and the cell viability was measured after 48 h. RPMI 1640 medium supplemented with 100 *μ*g/mL streptomycin, 100 U/mL penicillin, and 10% (v/v) FBS (all from Sigma, Munich, Germany) was used for the cultivation of MRC-5 cell line. Cells were maintained as a monolayer (1^·^10^4^ cells per well) in RPMI 1640 and grown in a humidified atmosphere of 95% air and 5% CO_2_ at 37°C. The extent of MTT reduction was measured spectrophotometrically at 540 nm using Tecan Infinite 200 Pro multiplate reader (Tecan Group Ltd, Männedorf, Switzerland), and the cell survival was expressed as a percentage of the control (untreated cells). Cytotoxicity was expressed as the concentration of the compound inhibiting cell growth by 50% (IC_50_).

### 2.7. DNA Binding Study

UV-Vis spectrophotometric titrations were performed by maintaining the concentration of the silver(I) complexes **1–3** constant and varying ctDNA concentration. The UV-Vis spectra were recorded in the range of 200–600 nm. The baseline was corrected by subtracting that of the Tris buffer. The samples were incubated for 5 min prior to measuring the spectra. From the obtained UV-Vis titration data, the binding constants (*K*_*b*_) were calculated using the following equation [28]:

(1) $$\begin{array}{c} \frac{\left[\text{DNA}\right]}{\varepsilon_{a}-\varepsilon_{f}}=\frac{\left[\text{DNA}\right]}{\varepsilon_{b}-\varepsilon_{f}}+\frac{1}{K_{b}\left(\varepsilon_{b}-\varepsilon_{f}\right)}, \end{array}$$

where *ε*_*a*_, *ε*_*b*_, and *ε*_*f*_ correspond to A_obs_/[complex] and the extinction coefficients of the complexes in the bound and free forms, respectively. In plots of [DNA]/(*ε*_*a*_ − *ε*_*f*_) versus [DNA], *K*_*b*_ is given by the ratio of the slope (1/(*ε*_*b*_ − *ε*_*f*_)) to the intercept (1/*K*_*b*_(*ε*_*b*_ − *ε*_*f*_)).

The competitive DNA-silver(I) complex binding experiments were carried out in the buffer (pH 7.4) by maintaining [DNA]/[EthBr] = 5, while increasing the concentration of the complexes **1–3**. Each sample solution was scanned in the wavelength range 525–800 nm with an excitation wavelength of 520 nm. The Stern-Volmer constants (*K*_*sv*_), which represent a measure of the binding propensity of the complexes to DNA, were calculated using the following equation [29]:

(2) $$\begin{array}{c} \frac{F_{0}}{F}=1+K_{q}\tau_{0}\left[\text{complex}\right]=1+K_{sv}\left[\text{complex}\right], \end{array}$$

where *F*_0_ and *F* are the fluorescence intensities in the absence and presence of the complexes, respectively. *K*_*q*_ stands for bimolecular quenching constant and *τ*_0_ (10^−8^ s) is the lifetime of the fluorophore in the absence of the quencher. The binding constants (*K*_*A*_) and apparent binding sites (*n*) can be calculated by using the following equation [28]:

(3) $$\begin{array}{c} \log\frac{\left(F_{0}-F\right)}{F}=\log\;K_{A}+n\;\log\left[\text{complex}\right], \end{array}$$

where *K*_*A*_ stands for the binding constant of the silver(I) complex with ctDNA and *n* stands for the apparent number of binding sites.

DNA interaction assay using gel electrophoresis has also been conducted according to the previously published procedure [17] using high molecular weight (HMW) genomic DNA isolated from *C. albicans* ATCC 10231. Briefly, DNA (50 ng/*μ*L final concentration) was incubated with 40, 100, and 400 *μ*M final concentration of silver(I) complexes **1**–**3** in Tris buffer, pH 8.5 in 50 *μ*L reaction volume. After 2 h incubation at 30°C, 10 *μ*L aliquots were taken, mixed with loading dye, and loaded on agarose gel. Control contained an appropriate volume of DMSO. DNA samples were run, 500 ng per lane, on 0.8% agarose gel with ethidium bromide (EthBr) against O'GeneRuler™ 1 kb Plus DNA Ladder (Thermo Scientific™) at 60 V for 1 h. Gels were visualized and analyzed using the Gel Doc EZ system (Bio-Rad, Life Sciences, Hercules, USA), equipped with the Image Lab™ Software.

### 2.8. BSA Binding Study

The protein binding study was performed by tryptophan fluorescence quenching experiments using BSA (13.1 *μ*M) in Tris buffer solution (pH 7.4). The quenching of the emission intensity of tryptophan residues of BSA at 352 nm was monitored using the increasing concentration of the complexes **1–3** (up to 464.9 *μ*M). Fluorescence spectra were recorded in the range 280–500 nm with an excitation wavelength of 275 nm. The corresponding binding constants of the complexes (*K*_*A*_) and apparent binding sites (*n*) were calculated as it was previously explained [28, 29].

## 3. Results and Discussion

### 3.1. Synthesis and Structural Features of Silver(I) Complexes **1–3**

**Figure 1:**
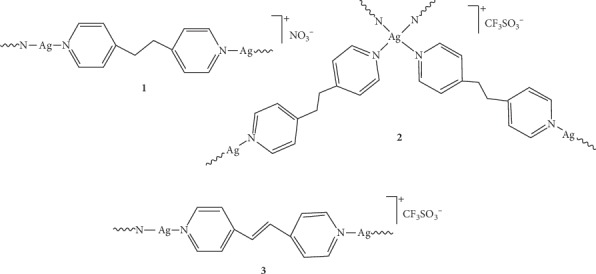
Structural representation of silver(I) complexes **1–3** analyzed in this study.

In the light of our recent results related to the promising antimicrobial activity of silver(I) complexes containing different aromatic *N*-heterocycles [17–21], two structurally different from those previously used, still belonging to general *N*-heterocycles class, with two pyridine rings linked by–CH_2_–CH_2_– and–CH**═**CH–groups, 1,2-bis(4-pyridyl)ethane (bpa) and 1,2-bis(4-pyridyl)ethene (bpe), respectively, were selected as ligands in this study, in order to obtain corresponding Ag(I) complexes and assess their biological activities. Three polynuclear silver(I) complexes, {[Ag(bpa)]NO_3_}_n_ (**1**), {[Ag(bpa)_2_]CF_3_SO_3_^.^H_2_O}_n_ (**2**), and {[Ag(bpe)]CF_3_SO_3_}_n_ (**3**) (Figure 1) were synthesized under different experimental conditions (solvents and molar ratio of the reactants) in respect to those previously reported in literature [24, 25]. The complexes were obtained in a high yield (>70%) by reacting AgX salt (*X* = NO_3_^−^ and CF_3_SO_3_^−^) with the corresponding *N*-heterocycle in 2 : 1 molar ratio in ethanol at ambient temperature. They are moderately soluble in water, but after the addition of a small aliquot of DMSO, the complexes **1–3** become completely soluble. Different spectroscopic techniques including ^1^H and ^13^C NMR, IR, and UV-Vis were used for the structural elucidation of these complexes and the obtained data were in accordance with those determined by spectroscopic and crystallographic measurements for the same complexes previously [24, 25]. Importantly, we have investigated the interaction of complexes **1–3** with ctDNA and BSA in correlation with the possible mechanism of their antimicrobial activity.

#### 3.1.1. Spectral Characterization

In the aromatic region, the ^1^H NMR spectra of the complexes **1–3**, measured in DMSO-*d*_*6*_, consist of two characteristic signals corresponding to eight protons on the two pyridine rings, with resonances slightly shifted downfield in respect to those for the same protons of the uncoordinated bpa and bpe ligands. Only weak displacement of the resonances of the silver(I) complexes with respect to the free ligands seems to be their specific spectroscopic feature in solution [30, 31]. The spectra of **1** and **2** are virtually identical as these complexes differ only in the type of counteranion. It is interesting to note that the differences in the chemical shifts of the H-3 and H-5 protons for the complexes **1**–**3** and these protons of the uncoordinated *N*-heterocycles are larger than those for H-2 and H-6 protons, which are adjacent to the pyridine nitrogen. Additionally, the aliphatic –CH_2_–CH_2_– group bridging two pyridine rings in **1** and **2** gave a singlet at 2.98 and 2.96 ppm, while in complex **3**, a singlet at 7.57 ppm can be assigned to the protons of –CH**═**CH– linker. The time-dependent ^1^H NMR spectra of the complexes **1–3** revealed that bpa and bpe ligands remain coordinated to the Ag(I) ion during 48 h.

The ^13^C NMR spectra of **1–3** are very similar to those of free bpa and bpe ligands. The most noticeable shifting (+0.95 ppm) is observed for C4 carbon in bpa after its coordination to Ag(I) ion in complex **1**, while the resonances for the remaining carbon atoms are nearly unaffected.

The IR spectra of the complexes **1–3** exhibit the bands due to the typical vibrations of the coordinated aromatic *N*-heterocycles as well as those of NO_3_^−^ and CF_3_SO_3_^−^ counteranions. In the IR spectrum of **1**, a very strong band at 1384 cm^−1^ is associated with asymmetric stretching vibration of uncoordinated NO_3_^−^ ion [32]. Complexes **2** and **3** having triflate as counteranion exhibit a few strong absorptions in the 1000–1300 cm^−1^ region [33, 34]. Thus, the bands at 1279, 1251, and 1024 cm^−1^ (**2**) and 1280, 1251, and 1025 cm^−1^ (**3**) are attributed to the asymmetric and symmetric stretching modes of the –SO_3_ group [33]. The splitting of the band due to the asymmetric stretching vibration of –SO_3_ group can be the consequence of its participation in hydrogen bonding interactions leading to its “pseudomonodentate” spectroscopic behavior [35]. Besides that, the two bands at 1223 and 1160 cm^−1^ (**2**) and 1222 and 1156 cm^−1^ (**3**) can be attributed to the symmetric and asymmetric stretching modes of –CF_3_ group of the triflate, respectively [34]. Additionally, in the IR spectrum of **2**, a broad absorption band at 3429 cm^−1^ is due to the stretching vibration of the OH group and confirms the presence of crystalline water molecules.

The wavelengths of maximum absorption for complexes **1–3** (*λ*_max_, nm) and molar extinction coefficients (*ε*, M^−1^ cm^−1^), determined immediately after their dissolution in DMSO, are given in the Materials and Methods section. As can be seen, the UV-Vis spectroscopic data for the silver(I) complexes are similar to those of the uncoordinated *N*-heterocycles, suggesting that the corresponding absorbance peaks in the complexes are a consequence of *π*⟶*π*^*∗*^ transitions in the corresponding ligand [36, 37].

### 3.2. Biological Evaluation of Silver(I) Complexes **1–3**

The microdilution assay was applied to screen the antimicrobial activity of silver(I) complexes **1–3** and the respective *N*-heterocyclic ligands towards various bacterial and fungal species (Table 1). The corresponding silver(I) salts used for the synthesis of the complexes (AgNO_3_ and AgCF_3_SO_3_) were evaluated previously for antimicrobial and antiproliferative potentials [20]. Among the bacterial strains, three Gram-positives (*Listeria monocytogenes*, *Enterococcus faecalis*, and *Staphylococcus aureus*) and four Gram-negatives (*Pseudomonas aeruginosa* PAO1, *Escherichia coli, Salmonella enteritidis*, *and Salmonella pullorum*) were considered. These bacteria are pathogens which are causative agents of skin and soft tissue infections, respiratory and urinary tract infections, and can be also associated with the use of different medical devices in hospitals (nosocomial infection). Among the fungi, four *Candida* species (*C. albicans*, *C. parapsilosis*, *C. glabrata*, and *C. krusei*) accounting for ≥95% of all candidemia were selected [38]. The antimicrobial activity of **1–3** and the ligands used for their synthesis, bpa and bpe, against the abovementioned strains is expressed as minimal inhibitory concentration (MIC, *μ*g/mL) and compared to the effect on the viability on the human normal fibroblast cell line (MRC-5) with the aim of evaluating the therapeutic potential of the complexes.

The investigated silver(I) complexes **1–3** exhibited moderate to good antibacterial activity with MIC values in the range of 12.5 to 250 *μ*g/mL, while the activity of bpa and bpe ligands towards the investigated bacterial strains is not significant (Table 1). With **1–3**, the best antibacterial activity was detected against the Gram-negative *E. coli* with MIC value being 12.5 *μ*g/mL. Among the complexes, it can be noticed that **1** with coordinated bpa and with nitrate counteranion exhibited the best antibacterial properties across the tested range of microorganisms. Moreover, this complex showed the best activity profile with low MIC values against bacteria, especially *E. coli* and *E. faecalis* and low cytotoxicity on the human normal fibroblast cell line MRC-5 with IC_50_ value being 40 *μ*g/mL (Table 1).

While being moderately active against the investigated bacterial strains (except *E. coli*), complexes **1–3** showed remarkable antifungal activity against the all tested *Candida* strains, with *C. parapsilosis* and *C. krusei* being the most sensitive (Table 1). Great sensitivity of *C. parapsilosis* is of special interest, as this strain was found to cause severe infections in neonates and patients in intensive care units [39]. The best activity against *C. parapsilosis* was observed for bpa-containing complex **2**, with a MIC value of 2.5 *μ*g/mL (Table 1). Nevertheless, both complexes **1** and **3** have a better therapeutic profile in the case of this strain, as their selectivity index was found to be 12.9. In general, all investigated silver(I) complexes exhibited moderate cytotoxicity on the normal human lung fibroblast cell line MRC-5, which is a desirable property for the possible application of these compounds as antifungal agents.

**Figure 2:**
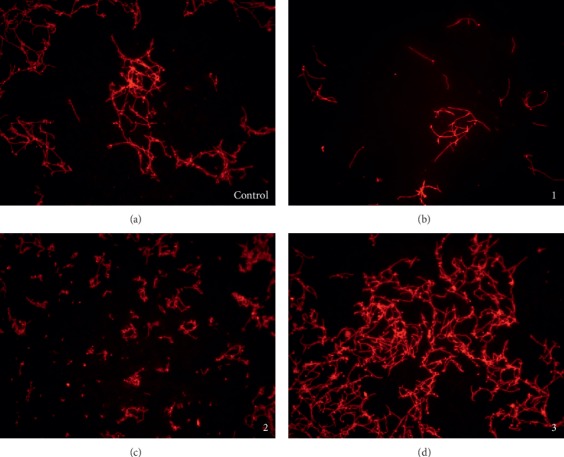
The effects of complexes **1–3** on *C. albicans* hyphae formation. *C. albicans* cells were treated with the MIC_80_ of complexes and DMSO treatment was used as the control. The images are taken by fluorescence microscope Olympus BX51 under 20× magnification.

Importantly, complex **2** was able to efficiently inhibit *C. albicans* morphological transformation from yeast to hypha form, which is one of the important pathogenic factors of these microorganisms. As observable in Figure 2, in the control (DMSO treated) sample, a large number of hyphae with branches were formed, while treatment with **2** resulted in the decreased number and length of hyphae. While complex **1** reduced the growth under tested conditions, both **1** and **3** did not significantly affect the hyphae formation (Figure 2).

The selectivity towards different *Candida* spp. in comparison to the bacteria was also previously shown by silver(I) complexes with 1,7- and 4,7-phenanthroline ligands [17, 21]. Moreover, at MIC doses, [Ag(NO_3_)(4,7-phen)]_n_ and [Ag(CF_3_COO)(4,7-phen)]_n_ (4,7-phen is 4,7-phenanthroline) totally prevented *C. albicans* filamentation and rescued the infected zebrafish of the lethal infection outcome [21]. Similarly, silver(I) complexes with substituted imidazoles, 2-amino-3-methylpyridine, pyridine-2-carboxaldoxime, and pyridine-3,5-dicarboxylate showed considerable *anti-Candida* activity and only moderate effectiveness against the tested bacterial strains [40–42]. On the other hand, a selective antibacterial activity, especially against *P. aeruginosa*, was observed for silver(I) complexes with *N*-heterocycles containing two nitrogen atoms within one ring, such as diazines (pyridazine, pyrimidine, and pyrazine), benzodiazines (phthalazine, quinazoline, and quinoxaline), and phenazine [18–20].

### 3.3. DNA Interactions

As previously reported, Ag(I) ion can induce the errors in DNA transcription processes, which might be responsible for the disturbance of the normal functionality of nucleic acids [29]. One of the most convenient techniques in DNA binding studies is UV-Vis spectrophotometry. The change in the UV-Vis spectra of the presently investigated complexes **1–3** in the presence of the increasing amount of ctDNA was investigated (Figure S1). As can be seen from the figure, with increasing concentration of ctDNA, the absorption bands of the complexes are affected and the hyperchromic effect was observed. The observed hyperchromic effect indicated that the silver(I) complexes **1–3** formed a noncovalent interaction with ctDNA via the external contact with the phosphate backbone (electrostatic binding) or groove (major or minor) binding [43–45]. Additionally, the intrinsic binding constants of the complexes (*K*_*b*_) can be calculated from a plot of [DNA]/(*ε*_*a*_ − *ε*_*f*_) versus [DNA] (Figure S1). From the calculated *K*_*b*_ values, it can be concluded that complex **1** binds stronger to the double-stranded beta-helix with respect to the remaining two complexes (Table 2). Nevertheless, the intrinsic binding constants for the studied silver(I) complexes are in accordance with those calculated for the previously reported silver(I) complexes, {[Ag(asp)(tpp)_3_(napr)](DMF)}, [Ag(Hsal)(tpp)_2_], [Ag(pHbza)(tpp)_2_], {[Ag(napr)(tpp)_3_](H_2_O)}, and [Ag(nim)(tpp)_2_], (Hnapr is naproxen, Hasp is aspirin, H_2_sal is salicylic acid, HpHbza is p-hydroxybenzoic acid, nim is nimesulide, and tpp is triphenylphosphine) [46], as well as for [Ag(pHbza)(tptp)_2_] and [Ag(nim)(tptp)_2_] (tptp is tri(*p*-tolyl)phospine) [46]. In order to investigate the spontaneity/nonspontaneity of the complex-DNA interaction, the Gibbs energy (ΔG) was calculated from the values of the binding constant (ΔG = -RTln*K*_b_). In all cases, ΔG has negative values, indicating the spontaneity of the interactions between the complexes **1–3** and ctDNA.

**Figure 3:**
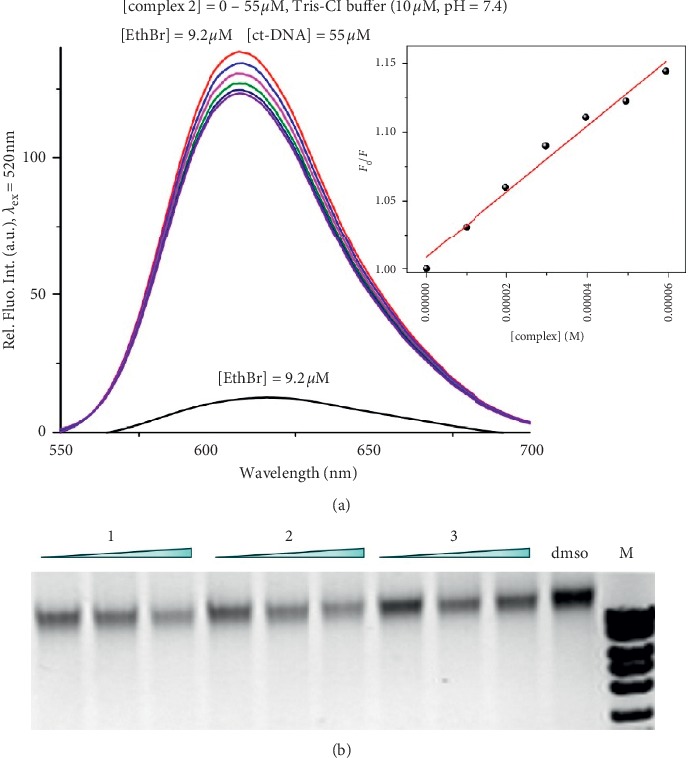
(a) Fluorescence emission spectra of EthBr-DNA system in the presence of an increasing amount of complex **2** (inserted graph: Stern-Volmer plots of relative EthBr-DNA fluorescence intensity *F*_0_/*F* versus [complex]). (b) *In vitro* interaction with *C. albicans* chromosomal DNA assessed by gel electrophoresis (increasing concentrations of complexes were 40, 100, and 400 *μ*M).

With the aim of gaining better information about the DNA binding affinities of the studied complexes **1–3**, the competitive binding experiments based on the displacement of EthBr from ctDNA were performed (Figures 3, S2, and S3). It was found previously that EthBr intercalates between adjacent base pairs in the DNA double helix leading to the enhancement of its fluorescence [29]. If a tested compound intercalates into DNA, a decrease in the fluorescence intensity of the EthBr-DNA system will be observed [21, 47]. Furthermore, binding of the tested compound to the EthBr-DNA can lead to the formation of a new nonfluorescence [complex]-EthBr-DNA system, causing the fluorescence quenching of EthBr-DNA [21]. As can be seen from Figures 3 and S2, the addition of the silver(I) complexes to the EthBr-DNA solutions caused the reduction in emission intensities. Considering the fact that the calculated binding constants (*K*_*A*_, Table 2) are significantly lower than those for EthBr (*K*_*A*_ = 2^.^10^6^ M^−1^) [47], the second assumption is a more reasonable explanation for the decrease in the fluorescence intensity of the EthBr-DNA system after the addition of the complexes **1–3**. The slight reduction of the emission of EthBr-DNA system was observable in gel electrophoresis assay as well, when **1–3** were added to genomic DNA from *C. albicans* in concentrations 40–400 *μ*M prior to EthBr staining, in comparison to the DMSO-treated sample (Figure 3(b)). Slightly higher interaction for **1** and **2** can be concluded in comparison to **3** under these conditions, especially at the highest concentration of complexes used.

As can be seen from Table 2, the values of Stern-Volmer constants (*K*_*sv*_) for the silver(I) complexes **1–3** are low and suggested that they bind to ctDNA through the nonintercalative mode. This can be also concluded from the percentage of hypochromism up to 12%. For instance, the percentage of hypochromism for lucigenin, a proven DNA intercalator, was found to be 50% [48]. From the *K*_*sv*_ values, it could be seen that bpa-containing complexes **1** and **2** show higher affinity for ctDNA in comparison with that of **3** having bpe as bridging ligand, which is in line with gel electrophoresis results (Figure 3(b)). Similar values of *K*_*sv*_ constants were obtained for the previously investigated silver(I) complexes by using the same methods [21, 49, 50]. From the values of *K*_*q*_ constants higher than the limiting diffusion rate constant of ctDNA (2^.^10^10^ M^−1^·s^−1^), it can be concluded that the interaction between silver(I) complexes **1–3** and this biomolecule is a static quenching process [47].

### 3.4. BSA Interactions

Serum albumin is the most abundant protein in the blood and plays an important role in the transport and delivery of many pharmaceuticals [51]. Therefore, the studies aimed at the binding of biologically active compounds with this protein provide useful information on their biodistribution, toxicity, and mechanism of action [52]. Bovine serum albumin (BSA) represents the structural analog of the human serum albumin (HSA) and is the most extensively studied serum albumin for metal complexes interactions [53]. It contains three fluorophores, namely, tryptophan, tyrosine, and phenylalanine; nevertheless, the intrinsic fluorescence of BSA is mainly due to tryptophan alone [54].

**Figure 4:**
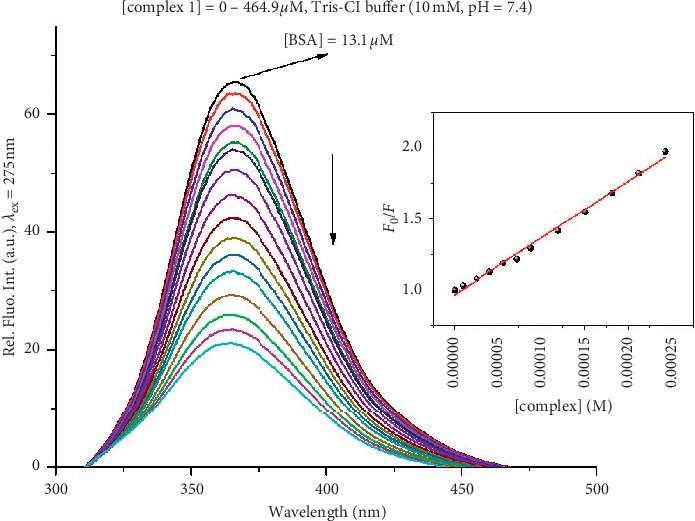
Fluorescence emission spectra of BSA in the presence of an increasing amount of complex **1**. Arrow shows the intensity changes upon increased concentrations of the complex. Inserted graph: Stern-Volmer plots of *F*_0_/*F* versus [complex].

The interaction of the complexes **1–3** with BSA was studied by fluorescence spectroscopy (Figures 4, S4, and S5). The addition of increasing amounts of the complexes to the BSA solution at a constant concentration resulted in a remarkable quenching of BSA fluorescence. This could be the consequence of the complexes binding to BSA, causing the changes in the tertiary structure of protein and tryptophan environment of BSA [55]. Furthermore, the decrease of fluorescence intensity of a fluorophore can be caused by energy transfer, excited-state reactions, molecular rearrangements, collision quenching, and ground-state complex formation [56]. To study the quenching mechanism induced by the presently investigated silver(I) complexes, the fluorescence quenching data were analyzed using the Stern-Volmer and Scatchard equations and the values of Stern-Volmer constants (*K*_*sv*_), quenching rate constants of biomolecule (*K*_*q*_), binding constants (*K*_*A*_), and number of binding sites per albumin (*n*) are reported in Table 3.

The *K*_*sv*_ values follow the order **3** > **2** > **1** (Table 3), indicating that the bpe-containing complex **3** has a higher affinity for BSA in comparison with those of **1** and **2,** both containing bpa linker (Figure 1). The *K*_*sv*_ value for **3** is similar to those obtained for silver(I) complexes with *N*-methyl-1,3,5-triaza-7-phosphaadamantane and tris(pyrazol-1-yl)methanesulfonate [29]. The quenching rate constant (*K*_*q*_) is found to be dependent on the probability of a collision between fluorophore and quencher and represents a measure of the exposure of tryptophan residues to the investigated complex [51]. The *K*_*q*_ values (Table 3) indicate that the complexes show good quenching ability of the BSA fluorescence, with complex **3** exhibiting the strongest (*K*_*q*_ = 2.76^.^10^12^ M^−1^·s^−1^). Moreover, the values of *K*_*q*_ constants are higher than the value of maximum diffusion collision quenching rate constant (2^.^10^10^ M^−1^·s^−1^), suggesting that the fluorescence quenching process of the silver(I) complexes **1–3** is mainly controlled by a static rather than a dynamic quenching mechanism [29, 47]. The values of the *K*_*A*_ constants for all complexes are optimal; they are high enough so that the complexes can bind to BSA to get transport, but not so high to prevent release from the BSA upon arrival to the target site [57]. The *n* values for the silver(I) complexes **1–3** suggest that there is only one binding site available on the investigated protein.

## 4. Conclusions

Synthesis of three polynuclear silver(I) complexes, {[Ag(bpa)]NO_3_}_n_ (**1**), {[Ag(bpa)_2_]CF_3_SO_3_^.^H_2_O}_n_ (**2**), and {[Ag(bpe)]CF_3_SO_3_}_n_ (**3**), bpa = 1,2-bis(4-pyridyl)ethane and bpe = 1,2-bis(4-pyridyl)ethene, described in this study allowed their biological activity assessment. The present study confirms that the nitrogen-containing heterocycles, bpa and bpe, in which two pyridine rings are linked by alkyl and alkenyl group, respectively, are effective bridging ligands between two Ag(I) ions forming exclusively polynuclear complexes with NO_3_^−^ and CF_3_SO_3_^−^ as counteranions. The investigated complexes showed selective and considerable activity against four different *Candida* spp. and Gram-negative bacterium *E. coli* in comparison to the other tested bacterial strains, being moderately toxic on the normal human lung fibroblast cell line MRC-5. The type of bridging ligand in the investigated complexes plays an important role in determining their biological activity and affinity to DNA and BSA biomolecules. Complex **1** shows the best therapeutic potential (the highest antimicrobial activity and the lowest cytotoxicity on the human normal fibroblast cell line MRC-5) across the tested range of microorganisms. Also, both bpa-containing complexes **1** and **2** have a higher affinity to ctDNA in comparison to **3** with bpe linker, while in the case of BSA, the latter complex shows the strongest quenching ability. All these findings should be taken into consideration in design of novel silver(I) complexes as potential antimicrobial agents.

## Figures and Tables

**Table 1:** Antimicrobial activity of silver(I) complexes **1**–**3** and the respective bpa and bpe ligands (MIC, *μ*g/mL) in comparison to their cytotoxic effect on the normal human fibroblast cell line MRC-5 (IC_50_, *μ*g/mL).

Test organism	Compound				
	1	2	3	bpa	bpe
*E. coli* NCTC 9001	12.5^a^	12.5	12.5	>500	250
*P. aeruginosa* PAO1	50	200	25	>500	500
*L. monocytogenes* NCTC 11994	50	50	25	>500	100
*S. aureus* ATCC 43300	50	200	100	>500	>500
*E. faecalis* ATCC 29212	12.5	25	25	>500	>500
*S. enteritidis* ATCC 13075	50	50	200	>500	>500
*S. pullorum* ATCC 13036	25	12.5	250	>500	>500
*C. albicans* ATCC 10231	6.25	6.25	25	125	200
*C. parapsilosis* ATCC 22019	3.1	2.5	3.1	250	125
*C. glabrata* ATCC 2001	6.25	12.5	25	>500	100
*C. krusei* ATCC 6258	6.25	3.1	6.25	125	200
MRC-5 cells	40^b^	30	40	300	220

^a^Results are given as mean of three independent measurements with a standard error being between 1% and 3%. ^b^Calculated IC_50_ values correspond to the concentrations required to inhibit 50% of the cell growth.

**Table 2:** Values of the binding constants of silver(I) complexes **1–3** with ctDNA.

Complex	UV-Vis titration		Fluorescent titration				
	*K*_*b*_ (M^−1^)	ΔG^o^ (kcal/mol)	*K*_*sv*_ (M^−1^)	Hypochromism (%)	*К*_*q*_ (M^−1^·s^−1^)	*K*_*A*_ (M^−1^)	*n*
**1**	6.65^.^10^5^	−7.9	(2.53 ± 0.21)^.^10^3^	11.3	2.53^.^10^11^	1.8^.^10^4^	1.40
**2**	4.58^.^10^5^	−7.7	(2.39 ± 0.51)^.^10^3^	12.0	2.39^.^10^11^	4.22^.^10^4^	0.80
**3**	6^.^10^5^	−7.8	(9.07 ± 0.70)^.^10^2^	3.8	9.07^.^10^10^	59.16	0.72

**Table 3:** Values of the binding constants of silver(I) complexes **1–3** with BSA.

Complex	*K*_*sv*_ (M^−1^)	Hypochromism (%)	*K*_*q*_ (M^−1^·s^−1^)	*K*_*A*_ (M^−1^)	*n*
**1**	(4.13 ± 0.99)^.^10^3^	65.65	3.45^.^10^11^	1.34^.^10^4^	1.14
**2**	(8.16 ± 0.16)^.^10^3^	76.10	8.16^.^10^11^	1.97^.^10^4^	1.10
**3**	(2.76 ± 0.05)^.^10^4^	81.13	2.76^.^10^12^	1.27^.^10^5^	1.30

## Data Availability

The spectroscopic data used to support the findings of this study are available from the corresponding author upon request.

## Supplementary Materials

Supplementary Materials Figure S1: (A) absorption spectra of the silver(I) complexes **1–3** in Tris buffer upon addition of DNA. The arrow shows the change of absorbance upon increasing concentration of DNA. (B) Plot of [DNA]/(*ε*_*a*_ − *ε*_*f*_) versus [DNA]. Figure S2: (A) fluorescence emission spectra of EthBr bound to DNA in the absence and presence of the silver(I) complexes **1** and **3** in Tris buffer at 25°C. The arrow shows the change upon increasing concentration of complex. (B) Stern-Volmer plots of relative EthBr-DNA fluorescence intensity *F*_0_/*F* versus [complex]. Figure S3: plots of log(*F*_0_−*F*)/*F* versus log[complex] for DNA interactions. Figure S4: fluorescence emission spectra of BSA in the presence of an increasing amount of complexes **2** and **3**. The arrow shows the intensity changes upon increased concentrations of the complex. Inserted graph: Stern-Volmer plots of *F*_0_/*F* versus [complex]. Figure S5: plots of log(*F*_0_ − *F*)/*F* versus log[complex] for BSA interactions.
