# Supplementary material for: Antimicrobial Activity and DNA/BSA Binding Affinity of Polynuclear Silver(I) Complexes with 1,2-Bis(4-pyridyl)ethane/ethene as Bridging Ligands
Source: Bioinorg Chem Appl. 2020 Apr 14;2020:3812050. doi: 10.1155/2020/3812050 (PMC7178511; doi:10.1155/2020/3812050)
Supplement: Supplementary Materials — Figure S1: (A) absorption spectra of the silver(I) complexes 1–3 in Tris buffer upon addition of DNA. The arrow shows the change of absorbance upon increasing concentration of DNA. (B) Plot of [DNA]/(εa − εf) versus [DNA]. Figure S2: (A) fluorescence emission spectra of EthBr bound to DNA in the absence and presence of the silver(I) complexes 1 and 3 in Tris buffer at 25°C. The arrow shows the change upon increasing concentration of complex. (B) Stern-Volmer plots of relative EthBr-DNA fluorescence intensity F0/F versus [complex]. Figure S3: plots of log(F0−F)/F versus log[complex] for DNA interactions. Figure S4: fluorescence emission spectra of BSA in the presence of an increasing amount of complexes 2 and 3. The arrow shows the intensity changes upon increased concentrations of the complex. Inserted graph: Stern-Volmer plots of F0/F versus [complex]. Figure S5: plots of log(F0 − F)/F versus log[complex] for BSA interactions. [file 3812050.f1.docx]

**TABLE OF CONTENTS**

| Figure S1: (A) Absorption spectra of the silver(I) complexes **1 – 3** in Tris buffer upon addition of DNA. Arrow shows the change of absorbance upon increasing concentration of DNA. (B) Plot of [DNA]/(*ε*_a_ – *ε*_f_) versus [DNA]. | S4 |
| --- | --- |
| Figure S2: (A) Fluorescence emission spectra of EthBr bound to DNA in the absence and presence of the silver(I) complexes **1** and **3** in Tris buffer at 25 ^o^C. Arrow shows the change upon increasing concentration of complex. (B) Stern-Volmer plots of relative EthBr-DNA fluorescence intensity F_0_/F *vs* [complex]. | S5 |
| Figure S3: Plots of log(F_0_ - F)/F *vs* log[complex] for DNA interactions. | S6 |
| Figure S4: Fluorescence emission spectra of BSA in the presence of an increasing amount of complexes **2** and **3**. Arrow shows the intensity changes upon increased concentrations of the complex. Inserted graph: Stern-Volmer plots of F_0_/F *vs* [complex]. | S7 |
| Figure S5: Plots of log(F_0_ - F)/F *vs* log[complex] for BSA interactions. | S8 |


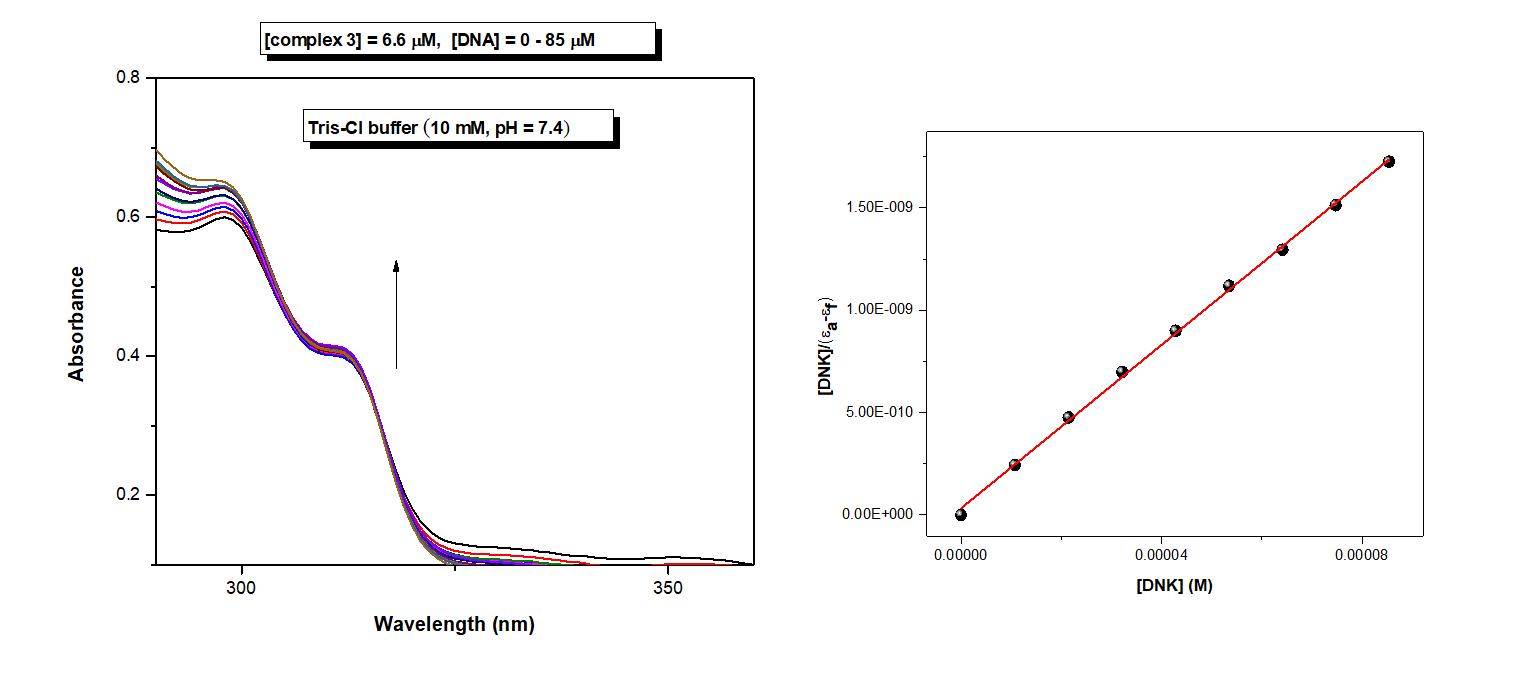

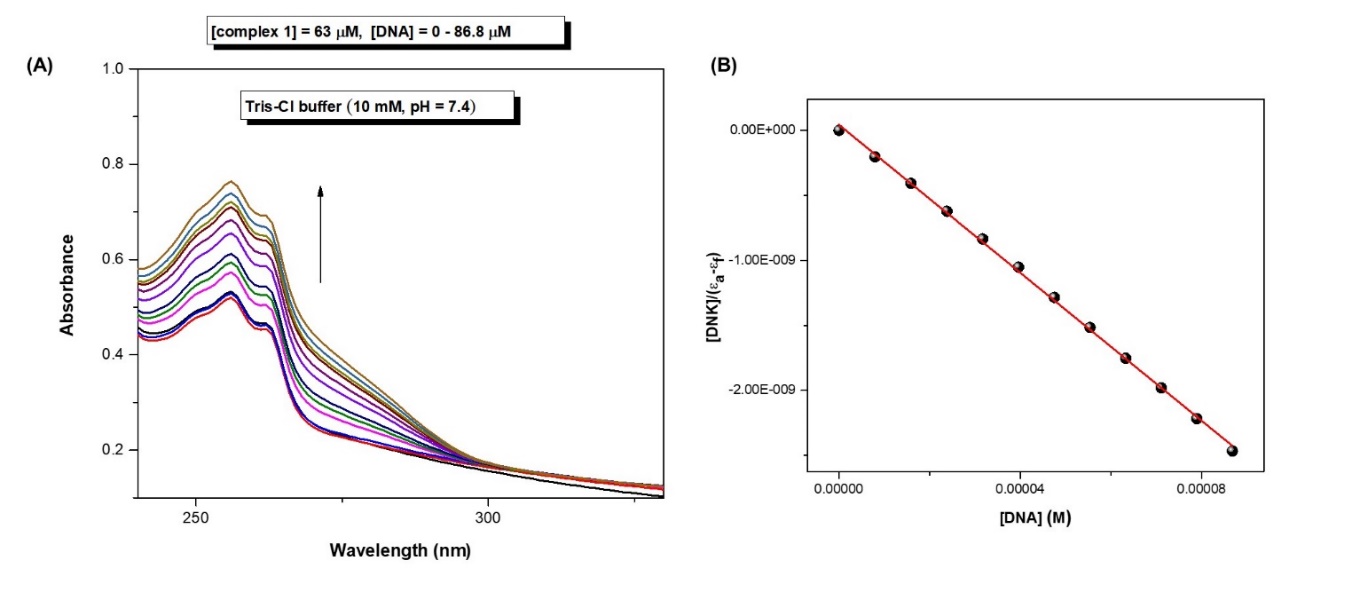


Figure S1: (A) Absorption spectra of the silver(I) complexes **1 – 3** in Tris buffer upon addition of DNA. Arrow shows the change of absorbance upon increasing concentration of DNA. (B) Plot of [DNA]/(*ε*_a_ – *ε*_f_) versus [DNA].


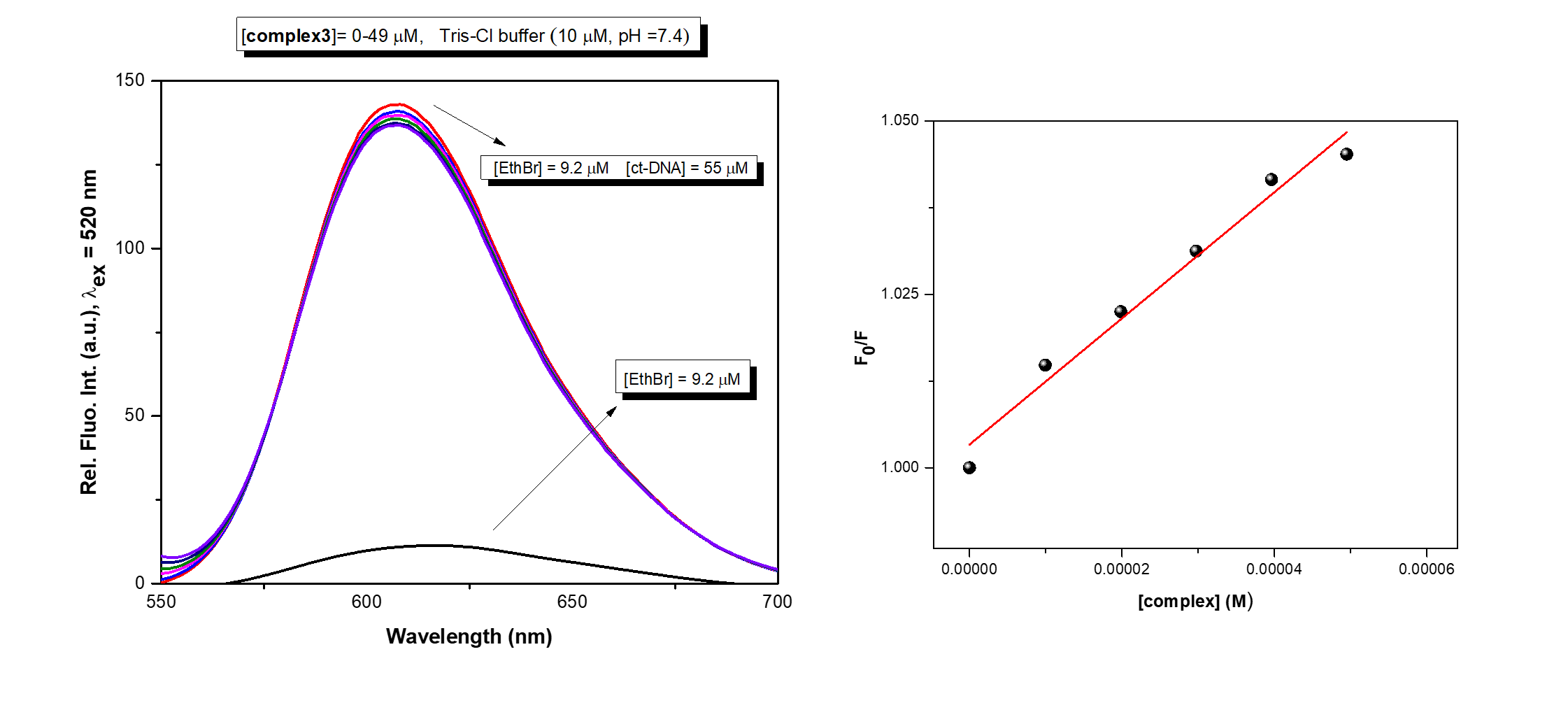

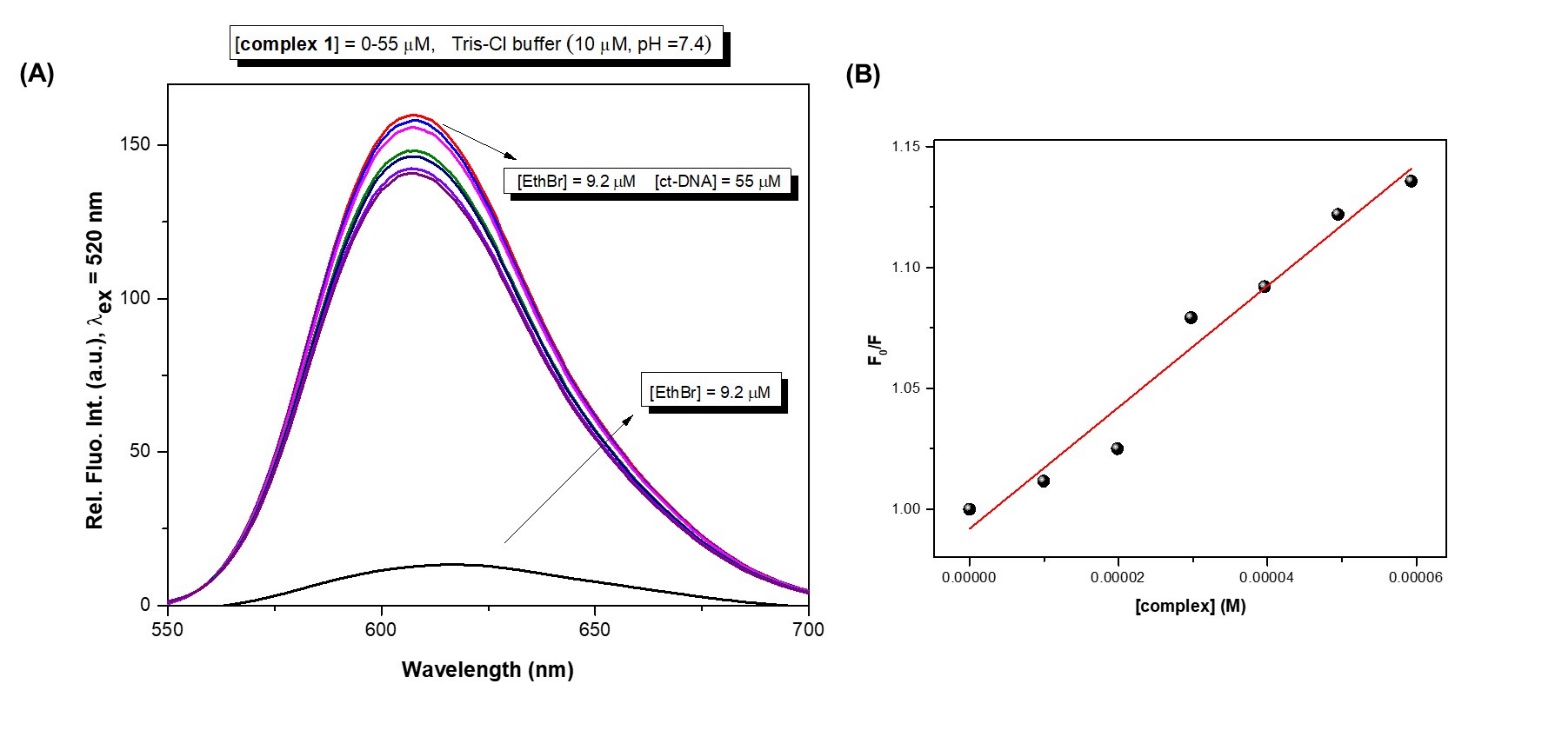


Figure S2: (A) Fluorescence emission spectra of EthBr bound to DNA in the absence and presence of the silver(I) complexes **1** and **3** in Tris buffer at 25 ^o^C. Arrow shows the change upon increasing concentration of complex. (B) Stern-Volmer plots of relative EthBr-DNA fluorescence intensity F_0_/F *vs* [complex].


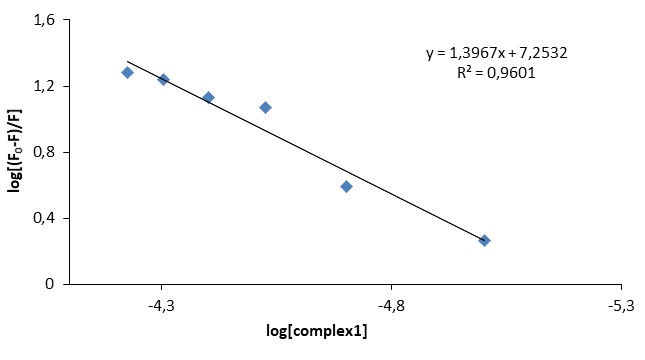


Figure S3: Plots of log(F_0_ - F)/F *vs* log[complex] for DNA interactions.

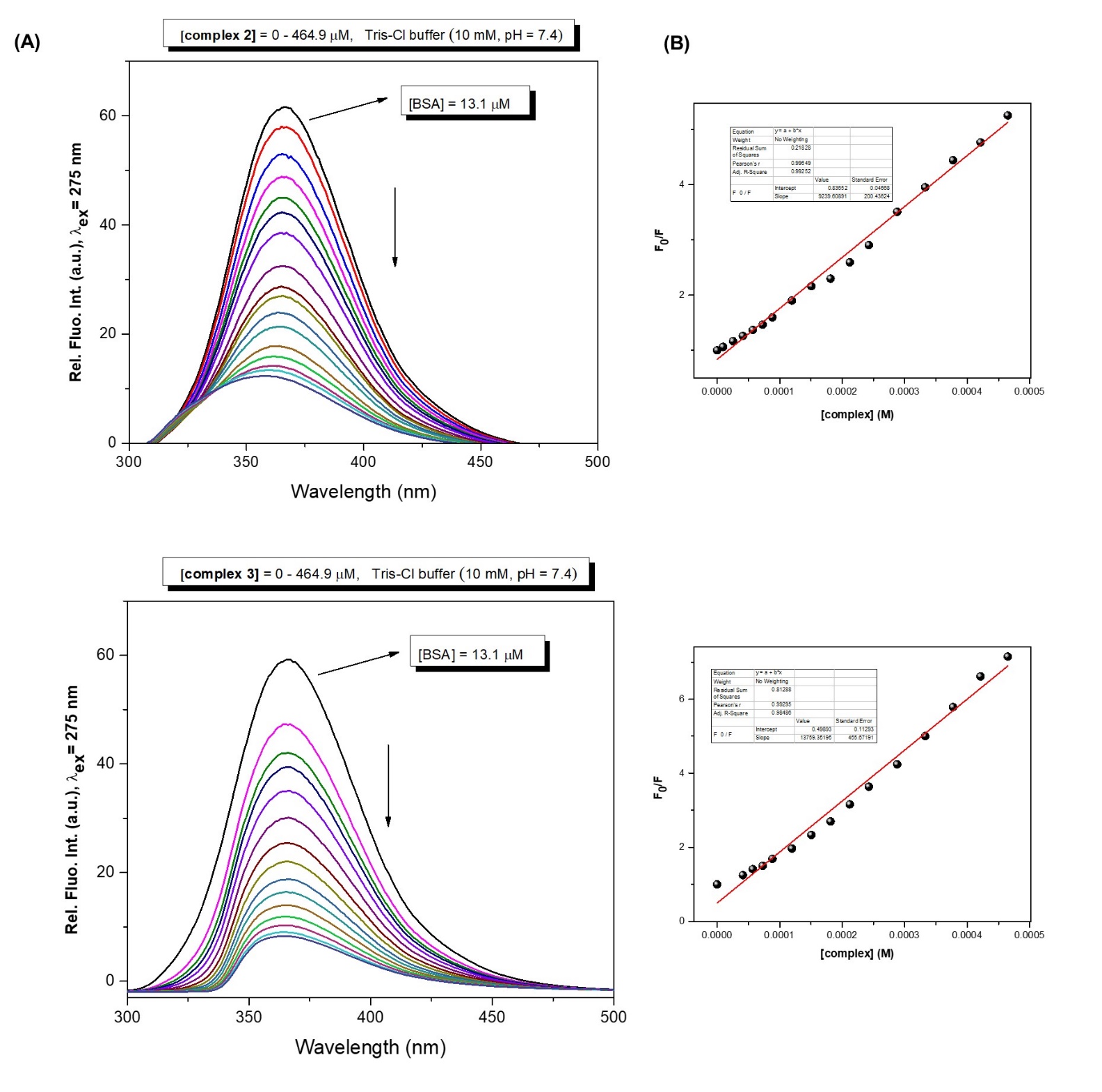


Figure S4: Fluorescence emission spectra of BSA in the presence of an increasing amount of complexes **2** and **3**. Arrow shows the intensity changes upon increased concentrations of the complex. Inserted graph: Stern-Volmer plots of F_0_/F *vs* [complex].


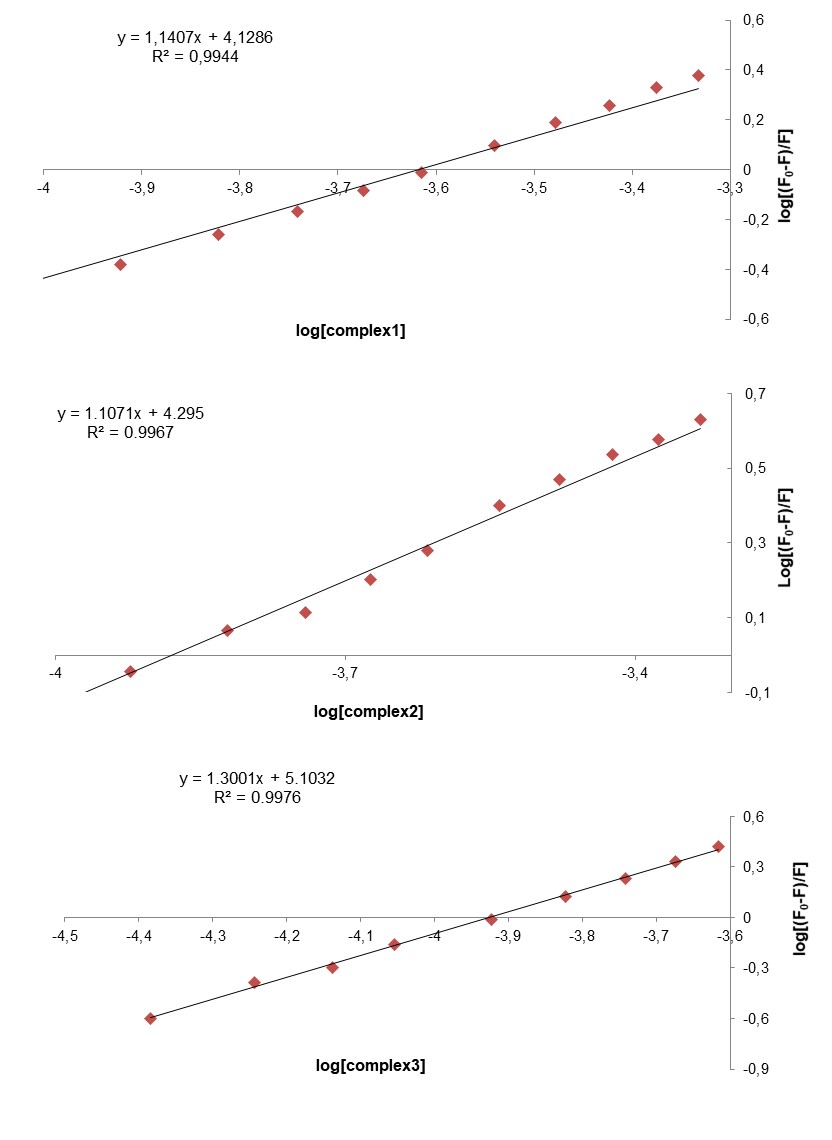


Figure S5: Plots of log(F_0_ - F)/F *vs* log[complex] for BSA interactions.
